# Effect of Alpha-Lipoic Acid on Rat Ventricles and Atria under LPS-Induced Oxidative Stress

**DOI:** 10.3390/antiox11040734

**Published:** 2022-04-08

**Authors:** Beata Skibska, Anna Goraca, Agnieszka Skibska, Andrzej Stanczak

**Affiliations:** 1 Department of Applied Pharmacy, Faculty of Pharmacy, Medical University of Lodz, 90-151 Lodz, Poland; andrzej.stanczak@umed.lodz.pl; 2 School of Cosmetology and Health Sciences, 94-016 Lodz, Poland; a.goraca@poczta.onet.pl; 3 Department of Biomolecular Chemistry, Medical University of Lodz, 92-215 Lodz, Poland; agnieszka.skibska@stud.umed.lodz.pl

**Keywords:** alpha-lipoic acid, oxidative stress, ventricles, atria, cardiac damage

## Abstract

Alpha-lipoic acid (α-LA) is a disulfide compound and one of the most effective antioxidants. Many studies have indicated positive effects of α-LA in the prevention of pathologic conditions mediated by oxidative stress, such as cardiovascular diseases. However, the therapeutic potential of α-LA for the heart has not been explored with regards to the ventricles and atria. The aim of our study was to evaluate the effects of α-LA on oxidative stress parameters and inflammation in the ventricles and atria of the heart in rats under LPS-induced oxidative stress. Wistar rats were divided into 4 groups: I—control (received 2 doses of 0.2 mL of 0.9% NaCl i.v., 0.5 h apart); II—α-LA (received 0.2 mL of 0.9% NaCl and 0.5 h later received α-LA 60 mg/kg b.w. i.v.); III—lipopolysaccharide (LPS) (received 0.2 mL of 0.9% NaCl and 0.5 h later received LPS 30 mg/kg b.w. i.v.); and IV—LPS + LA (received LPS 30 mg/kg b.w. i.v. and 0.5 h later received α-LA 60 mg/kg b.w. i.v.). Five hours later, the rats were euthanized. The hearts were surgically removed and weighed to estimate heart edema. The ventricular and atrium tissue was isolated to measure levels of TNF-α, IL-6, superoxide dismutase (SOD), thiobarbituric acid reactive substances (TBARS), hydrogen peroxide (H_2_O_2_), total sulfhydryl groups (-SH), total glutathione (tGSH), reduced glutathione (GSH), glutathione disulfide (GSSG), and the GSH/GSSG ratio. LPS significantly increased TNF-α, IL-6, TBARS, and H_2_O_2_ levels and decreased SOD, -SH groups, tGSH, the GSH/GSSG ratio, and GSH levels in rat ventricles and atria while α-LA administered after the injection of LPS significantly decreased TNF-α, IL-6, TBARS, and H_2_O_2_ levels. α-LA also increased SOD and -SH group levels and ameliorated the glutathione redox status when compared to the LPS group. Our data suggest that α-LA administration 30 min after LPS infusion may effectively prevent inflammation and oxidative stress in the ventricles and atria.

## 1. Introduction

Oxidative stress is caused by overexpression of reactive oxygen species (ROS), including hydroxyl radicals, superoxide anion, and hydrogen peroxide (H_2_O_2_), and has been documented to play a crucial role in the pathogenesis of cardiovascular diseases [1,2,3,4]. 

The heart has one of the highest oxygen consumption rates in the body and is highly susceptible to ROS [5]. Atria and ventricles are particularly susceptible to oxidative stress [6].

In many experimental models, oxidative stress and inflammation are induced by lipopolysaccharide (LPS) (a wall component of Gram-negative bacteria) [7]. 

Multiple mechanisms are implicated in LPS-induced cardiac damage. These involve different cell types and anatomical structures in the heart, including cardiomyocytes, and vasculature. Excessive levels of ROS can damage cardiac tissue through oxidation of lipids, proteins, and DNA, compromising mitochondrial structure and performance, enzyme function, and intracellular signaling [8]. Consequently, this contributes to cardiac remodeling, apoptosis, and eventually necrosis [9].

Studies suggest that oxidative stress can increase heart susceptibility to arrhythmias, and their prevention or attenuation can abolish proarrhythmic factors [10]. A variety of experimental studies have indicated that oxidative stress is implicated in the pathogenesis of heart failure (HF), atrial fibrillation (AF), and ischemic heart disease (IHD) [11,12,13]. AF development and perpetuation depend on the electrophysiological and structural substrates of atria [14]. 

Oxidative stress is regarded as an initiator of inflammation and a consequence of inflammatory responses. Inflammation causes myocardial dysfunction, which leads to endothelial dysfunction. Inflammatory markers, such as interleukin 6 (IL-6) and tumor necrosis factor-α (TNF-α), are released from cells of failing myocardium and from endothelial cells [15,16].

Experimental evidence suggests that antioxidant treatment may induce beneficial effects on cardiovascular damage caused by inflammation and oxidative stress [17,18,19].

One of the most important antioxidants is alpha-lipoic acid (α-LA). It is a disulfide compound that can quench free radicals and indirectly recirculate cellular antioxidants. α-LA is used as a cofactor of the mitochondrial respiratory enzymes pyruvate dehydrogenase and α-ketoglutarate dehydrogenase [20]. Many studies have reported the effectiveness of α-LA in the prevention of oxidative stress-mediated pathologic conditions [21,22]. In addition, α-LA exhibits anti-inflammatory effects by downregulating the expression of redox-sensitive proinflammatory proteins, including TNF-α, Il-6, and inducible nitric oxide synthase [23,24]. α-LA exhibits significant antioxidant activity by protecting against oxidative damage and inflammation in several diseases, including cardiovascular disorders.

The antioxidant properties of α-LA were demonstrated in rats submitted to sepsis. α-LA was able to reduce oxidative stress in the heart and other organs, including the liver and kidney, after sepsis [25]. α-LA attenuated the oxidative stress response in experimental models of heart failure and muscle injury [26]. It prevented the development of cardiac hypertrophy and both attenuated and delayed the onset of diastolic dysfunction.

The therapeutic effect of α-LA was associated with decreased TBARS levels and nucleic acid oxidation, and α-LA prevented the expression of adhesion molecules in the cardiac vascular endothelium [27].

Studies have demonstrated an association between the supplementation of α-LA and a decreased cardiovascular risk in humans and rats [28,29,30].

Because of its antioxidant abilities, α-LA could be considered a therapeutic factor for the treatment of cardiovascular diseases, induced by oxidative stress. 

Previously, we demonstrated that the antioxidant α-LA treatment reduced oxidative stress and prevented inflammation in the heart [31]. Until now, the antioxidant effect of α-lipoic acid within various areas of the heart has not been demonstrated.

However, we wanted to identify differences between the atria and the ventricles regarding parameters of inflammation and oxidative stress, and whether α-LA acts in a similar way in these two areas of the heart. This provides an insight into the structures of the heart and prompts an investigation, in more detail, into the mechanisms of action of α-LA in different areas of the heart. 

Therefore, the current study aimed to investigate changes in the ventricles and atria caused by LPS-induced oxidative stress and also investigate the potential benefits of α-LA for the ventricles and atria.

## 2. Materials and Methods

### 2.1. Chemicals and Reagents

α-Lipoic acid and lipopolysaccharide (*Escherichia coli* LPS 026:B6; lyophilized powder chromatographically purified by gel filtration, protein content < 1%) were purchased from Sigma Aldrich Chemical Co. (St. Louis, MO, USA). α-Lipoic acid was solubilized in 3 M NaOH-supplemented physiologic solution and buffered to 7.35–7.45 pH by adding 0.5% HCl. Commercial rat kits were used to evaluate TNF-α; IL-6; SOD; TBARS; total, reduced, and oxidized glutathione; and GSH/GSSG ratio assays.

All other reagents were provided by Sigma Aldrich Chemical Co. (St. Louis, MO, USA) or POCH (Gliwice, Poland).

### 2.2. Animals and Treatments

This study was approved by the Ethics Committee of the Medical University of Lodz, Poland, permission number 7/LB699/2014, and was conducted according to the guidelines of the Declaration of Helsinki. 

This experiment was performed on male Wistar rats weighing 260–280 g. The animals were acquired from the Medical University of Lodz animal quarters and were housed 3–4 per cage in standard laboratory conditions: 12 h light/dark cycles at a temperature of 22 ± 2 °C and 55 ± 5% air humidity. The rats were given free access to a standard laboratory diet and water ad libitum. After 1 week of acclimatization, 32 rats were randomly divided into 4 groups (*n* = 8), as follows: 

Group I—received 2 doses of 0.2 mL of 0.9% NaCl, 0.5 h apart, and served as a control. 

Group II—received 0.2 mL of 0.9% NaCl and 0.5 h later received α-LA (60 mg/kg b.w.). 

Group III—received 0.2 mL of 0.9% NaCl and 0.5 h later received LPS (30 mg/kg b.w.).

Group IV—received LPS (30 mg/kg) and 0.5 h later received α-LA (60 mg/kg b.w.). 

Intravenous administration of LPS represents an in vivo model of systemic inflammation in humans. All compounds were injected into the tail vein and after this administration, each group of animals was observed for a period of 5 h. The animals were sacrificed under ketamine/xylazine anesthesia. 

The heart was removed, rinsed with cold isotonic saline, and the atrium and ventricle were excised. Then, ventricular and atrium tissue was stored at −80 °C for preparation of the homogenates used for biochemical assessment, including oxidative stress markers and inflammatory cytokines.

### 2.3. Homogenization of Tissues

First, 50 mg portions of cardiac tissue from the left ventricle and/or left atrium were cut into small pieces and homogenized in 2 mL of ice-cold PBS with protease inhibitors (in a glass homogenizer). The resulting suspension was subjected to two freeze-thaw cycles to further break cell membranes. The homogenates were centrifuged for 5 min at 5000× *g* at 4 °C. After centrifugation, the supernatant was separated and applied to determine the oxidative stress markers and inflammation-associated cytokines. 

### 2.4. Inflammatory Markers

#### 2.4.1. Determination of TNF-α and IL-6

The commercial rat ELISA kit (R&D Systems, Minneapolis, MN, USA) was used to evaluate TNF-α whereas for IL-6, the Cloud-Clone Corp. (Houston, TX, USA) High Sensitive Enzyme-linked Immunosorbent Assay Kit (HEA079Ra, Houston, TX, USA) was used.

These markers were measured according to the manufacturer’s instructions. TNF-α and IL-6 concentrations were determined from the standard curves and expressed in pg/mg protein. 

#### 2.4.2. Changes in the HW (Heart Weight)/BW (Body Weight) Ratio

The hearts were rinsed with cold 0.9% NaCl, dried, and weighed to estimate heart edema. The ratios of heart weight to body weight (HW/BW) were measured and used as an index of heart edema.

### 2.5. Oxidative Stress Markers

#### 2.5.1. Determination of Superoxide Dismutase (SOD)

The enzymatic activity of SOD was determined using a commercially available ELISA test kit containing a monoclonal antibody specific for rat SOD (Cloud-Clone Corp., No. SEB960Ra, Houston, TX, USA), following the manufacturer’s instructions.

#### 2.5.2. Determination of Lipid Peroxidation Products (Thiobarbituric Acid Reactive Substances—TBARS)

Thiobarbituric acid reactive substances (TBARS) were measured in cardiac tissue from the ventricle and atrium using the Cayman Chemical TBARS Assay Kit (Item No. 10009055-96, Minneapolis, MN, USA), following the manufacturer’s instructions.

#### 2.5.3. Estimation of Tissue Hydroxyl Peroxide Content (H_2_O_2_)

Hydrogen peroxide (H_2_O_2_) generation in homogenates was measured using the HRP/HVA systems. First, 50 mg of frozen tissue fragments were homogenized with 2 mL of 1.15% KCl. Then, 10 μL of homogenate were placed in 2 Eppendorf tubes. In one tube, the reaction mixture for the calibration curve of H_2_O_2_ was added, which contained phosphate-buffered saline (PBS) (pH 7.0) and horseradish peroxidase (HRP) (1 U/mL) containing 400 μmol homovanillic acid (HVA). In the second tube, we placed PBS and 1 U/mL HRP to determine H_2_O_2_.

Both tubes were simultaneously incubated for 60 min at 37 °C. Subsequently, PBS and 0.1 M glycine-NaOH buffer (pH 12.0) with 25 mM EDTA were added to each Eppendorf tube to stop the enzymatic reaction. Excitation was set at 312 nm and emission was measured at 420 nm (Perkin Elmer Luminescence Spectrometer, LS-50, Norwalk, CT, USA). Readings were converted to the H_2_O_2_ concentration using the regression equation prepared from 3 series of calibration experiments with 10 increasing H_2_O_2_ concentrations (range 10–1000 μM).

#### 2.5.4. Measurement of Total Sulfhydryl Groups (-SH)

Total sulfhydryl groups measurement was performed using Ellman’s method [32]. As a chromogen, to measure thiol levels, Ellman’s reagent (5,5′-dithiobis-(2-nitrobenzoic) acid, DTNB) was used. The absorbance of the obtained solution was measured at 412 nm using the Perkin Elmer Luminescence Spectrometer LS-50 (Norwalk, CT, USA). The total concentration of thiol groups was calculated from the regression equation prepared from 3 repeats of increasing concentrations of glutathione (2–200 µM). Concentrations of -SH groups were expressed as μM.

#### 2.5.5. Total Glutathione (tGSH), Reduced Glutathione (GSH), Oxidized Glutathione (GSSG), and Total Glutathione/Oxidized Glutathione Assays (GSH/GSSG)

To measure total (GSH total) and oxidized state (GSSG) of glutathione, the tissue sample was divided in two parts. Both parts were homogenized in the same way (in buffer containing 50 mM phosphate and 1 mM EDTA). The only difference was the addition of a scavenger (2-vinylpyridinium) to the GSSG sample. Scavenger complexes with GSH and prevents it from reacting with the dye and interfering with the measurement of GSSG.

The total glutathione was analyzed using the Cayman Chemical Glutathione Assay kit (Item No. 703002-96, Ann Arbor, MI, USA). All steps were performed according to the manufacturer’s instructions. Before assaying the total glutathione level, the pH of the samples was increased with TEAM Reagent. Standards and samples were added to the wells together with Assay Cocktail (mixture of MES Buffer, Cofactor, Enzyme, and DTNB). After the incubation step, the total glutathione level was measured.

The reduced glutathione (GSH), oxidized glutathione (GSSG), and total glutathione/oxidized glutathione ratio (GSH/GSSG) were determined using the Abnova GSH/GSSG Assay kit (Item No. KA3779, Minneapolis, MN, USA). All steps were performed according to the manufacturer’s instructions. Standards and samples were added to the wells together with Working Reagent (mixture of Assay Buffer, GR Enzyme, NADPH, and DTNB) and the GSH/GSSG level was measured.

### 2.6. Statistical Analysis

The data are presented as mean ± SEM from 8 animals in each group. The statistical significance was evaluated by ANOVA followed by the Duncan’s multiple range test as post-hoc. Values of *p* < 0.05 were adopted as significant. 

## 3. Results

### 3.1. Assessment of TNF-α and IL-6

**Figure 1:**
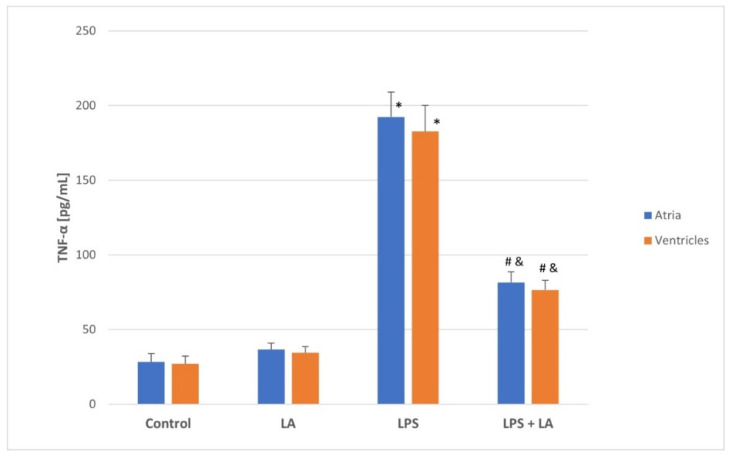
Effect of LA, LPS, and LPS + LA on TNF-α levels in ventricular and atrial homogenates. Results are shown as mean ± S.E.M. *n* = 8. * *p* < 0.05 vs. control, LA; # *p* < 0.01 vs. LPS; & *p* < 0.05 vs. control, LA. LA—alpha lipoic acid, LPS—lipopolysaccharide.

**Figure 2:**
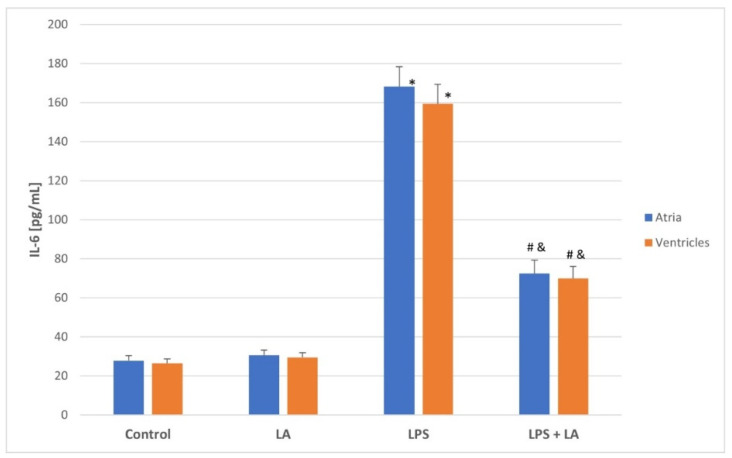
Effect of LA, LPS, and LPS + LA on Il-6 levels in ventricular and atrial homogenates. Results are shown as mean ± S.E.M. *n* = 8. * *p* < 0.05 vs. control, LA; # *p* < 0.01 vs. LPS; & *p* < 0.05 vs. control, LA. LA—alpha lipoic acid, LPS—lipopolysaccharide.

As shown in Figure 1 and Figure 2, the LPS challenge caused a marked increase in the levels of TNF-α and IL-6 in both the atria and ventricles compared to the control and LA group (*p* < 0.05). TNF-α and IL-6 levels appeared to be significantly reversed after LA treatment (*p* < 0.01) in both the ventricles and atria.

There were no significant differences in the TNF-α and IL-6 levels between the atria and ventricles in each group. As expected, administration of LA after LPS significantly reversed the inflammatory changes in the rat ventricles and atria.

### 3.2. Evaluation of Heart Edema

In LPS-induced rats, the HW/BW ratio was markedly higher when compared to control rats (*p* < 0.01), which implied progression of advanced heart edema. Administration of LA after LPS challenge ameliorated LPS-induced heart edema (*p* < 0.001) (Table 1). The results suggest that α-LA attenuated heart edema.

### 3.3. SOD Activities

**Figure 3:**
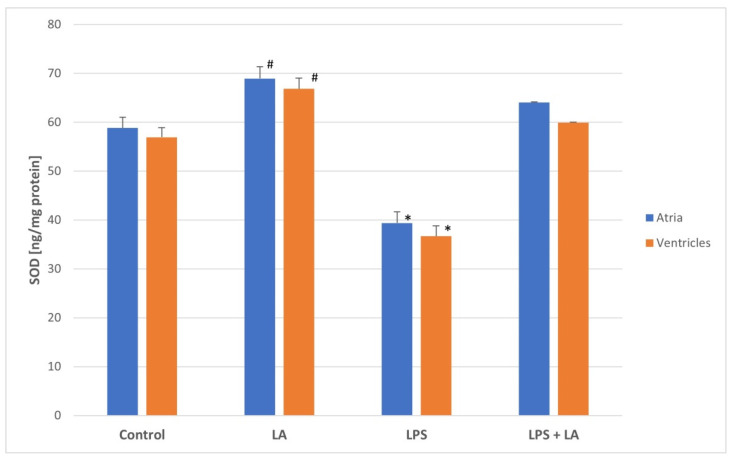
Effect of LA, LPS, and LPS + LA on SOD activities in ventricular and atrial homogenates. Results are shown as mean ± S.E.M. *n* = 8. * *p* < 0.05 vs. control, LA, LPS + LA; # *p* < 0.01 vs. control. LA—alpha lipoic acid, LPS—lipopolysaccharide.

To determine if a redox change systemically occurs as a result of LA administration, SOD activity in the ventricles and atria was measured. As shown in Figure 3, SOD activity was significantly decreased in the LPS group (both in the ventricles and atria) while LA treatment significantly increased SOD enzyme activities compared to the LPS group (*p* < 0.05).

Although the SOD values were similar between the control and LA groups, slightly higher SOD values were observed in the LA group than in the control group (*p* < 0.01).

There were no significant differences in the SOD activity between the rat atria and ventricles in each group.

### 3.4. TBARS Evaluation

TBARS was used to quantify the degree of lipid peroxidation in ventricular and atrial tissues.

TBARS values differed in the LPS groups between the ventricles and atria, indicating that the ventricles generated higher lipid peroxidation compared to the atria. 

Statistically significantly higher TBARS levels were found in both LPS groups (in both the ventricles and atria) as compared to the control group (*p* < 0.05). 

**Figure 4:**
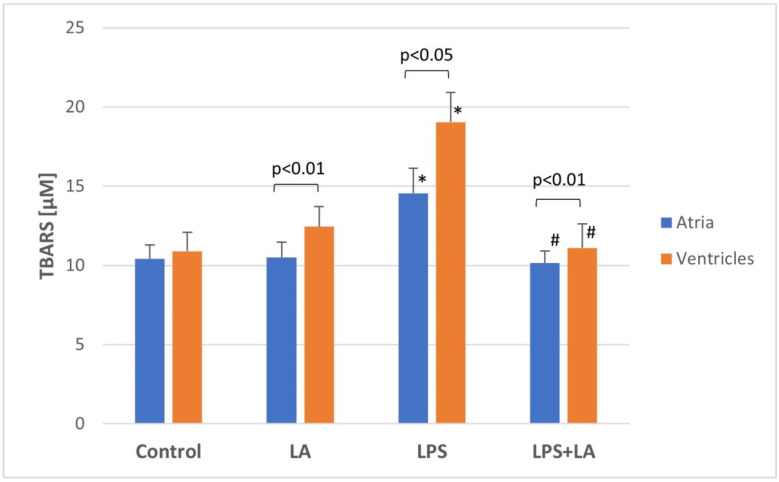
Effect of LA, LPS, and LPS + LA on TBARS levels in ventricular and atrial homogenates. Results are shown as mean ± S.E.M. *n* = 8. * *p* < 0.05 vs. control, LA; # *p* < 0.01 vs. LPS. LA—alpha lipoic acid, LPS—lipopolysaccharide.

The TBARS level in the LPS + LA groups was statistically significantly lower than in the LPS groups (*p* < 0.01). In conclusion, there was a significant increase in the TBARS level in the LPS group, which was restored after LA treatment (Figure 4) (in both the ventricles and atria).

### 3.5. Effect of LA on LPS-Induced H_2_O_2_ Levels

**Figure 5:**
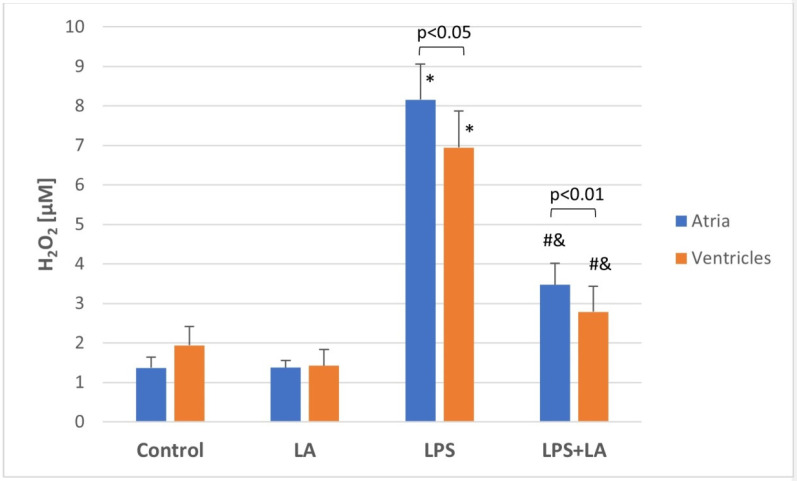
Effect of LA, LPS, and LPS + LA on H_2_O_2_ generation in ventricular and atrial homogenates. Results are shown as mean ± S.E.M. *n* = 8. * *p* < 0.05 vs. control, LA; # *p* < 0.01 vs. LPS; & *p* < 0.05 vs. control, LA. LA—alpha lipoic acid, LPS—lipopolysaccharide.

To investigate the endogenous generation of ROS, H_2_O_2_ was measured in rat ventricular and atrial homogenates. As shown in Figure 5, LPS caused a marked increase in the H_2_O_2_ level in rat tissues when compared to the control and LA groups (*p* < 0.05). LA effectively inhibited LPS-induced ROS generation in ventricular and atrial homogenates, compared with the LPS group (*p* < 0.01). This effect indicates mitigation of oxidative damage to the heart by LA. 

The H_2_O_2_ values differed in the LPS group between the ventricles and atria, indicating that the atria showed a higher concentration of ROS compared to the ventricles. 

### 3.6. Assessment of Sulfhydryl Groups (-SH) 

**Figure 6:**
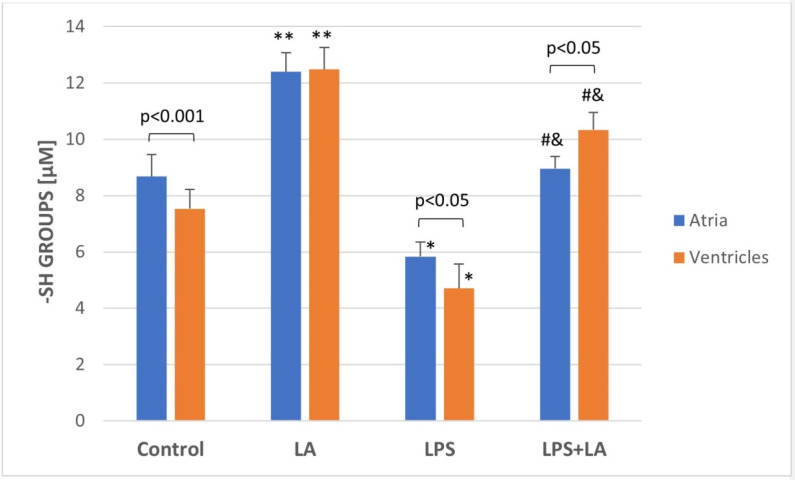
Effect of LA, LPS, and LPS + LA on sulfhydryl groups (-SH) content in ventricular and atrial homogenates. Results are shown as mean ± S.E.M. *n* = 8. * *p* < 0.05 vs. control, LA; # *p* < 0.01 vs. LPS; and & *p* < 0.05 vs. control, LA; ** *p* < 0.05 vs. control. LA—alpha lipoic acid, LPS—lipopolysaccharide.

As shown in Figure 6, administration of LPS significantly decreased the level of -SH groups in ventricular and atria homogenates compared with the control and LA groups (*p* < 0.05). Administration of LA 30 min after LPS infusion resulted in a significant increase in the -SH group content when compared to the LPS group (*p* < 0.01). 

Interestingly, the results demonstrated significant increases in the -SH groups content in the ventricular and atrial homogenates after administration of LA compared to the control group (*p* < 0.05). The -SH values differed in the LPS and LPS + LA groups between the ventricles and atria. In the atria, the -SH content in the LPS group was higher than in the ventricles. However, in the LPS + LA group, the -SH content in the atria was lower than in the ventricles.

### 3.7. Assessment of Glutathione

Oxidative stress in the tissue involves the GSH system. Therefore, levels of total GSH (tGSH), oxidized GSH (GSSG), reduced GSH, and the GSH/GSSG ratio were measured. Table 2 shows the levels of tGSH, GSH, GSSG, and the GSH/GSSG ratio in ventricular homogenates of the control and experimental groups of rats, whereas Table 3 presents these parameters in atria homogenates.

In the LPS group, the GSH level (an indicator of antioxidant capacity) was significantly decreased compared to the control and LA groups (*p* < 0.01) and it was associated with increased GSSG (*p* < 0.01). The GSH/GSSG ratio, which reflects the intracellular glutathione redox balance, was also significantly decreased in the LPS group compared to the control and LA groups (*p* < 0.05). In the LPS + LA group, both the reduced GSH level and the GSH/GSSG ratio were increased compared to the LPS group (*p* < 0.001).

There were no significant differences in the glutathione levels in the atria and ventricles in each group.

## 4. Discussion

In recent years, antioxidants have become elements of therapies aiming at improving cardiac damage in the prevention of cardiovascular diseases (CVDs), such as heart failure, atrial fibrillation, and ischemic heart disease. 

The properties of α-LA, in particular its antioxidant potential, have been widely revised in many studies [33,34,35,36]. There is evidence showing that α-LA has therapeutic activity in the regression of oxidative stress associated with CVDs [37,38,39]. 

The aim of the present study was to analyze and compare changes in the ventricles and atria during the development of acute inflammation and oxidative stress induced by LPS in rats and demonstrate the pivotal action of α-lipoic acid as an antioxidant. 

Oxidative stress is associated with overproduction of ROS and reduced cardiac antioxidant capacity [40,41]. Due to its high toxicity, LPS is considered the most potent initiator for activating inflammatory mediators and factors of oxidative stress [42]. Oxidative stress can disrupt cardiac mitochondrial activity through induction of damage to mitochondrial DNA, lipids, and proteins. It can also impair cardiomyocytes and activate several mitochondria apoptotic signaling pathways [43]. 

Since LPS can induce inflammation and leads to the development of oxidative stress in rats, this experimental model was used to induce cardiac damage. 

In the present study, the administration of LPS to rats resulted in the development of inflammation and oxidative stress in the ventricles and atria. This effect was indicated by an increase in the TBARS, H_2_O_2_, TNF-α, and IL-6 concentration and a decrease in the concentration of total sulfhydryl groups, SOD, and GSH. In LPS-induced rats, the HW/BW ratio was markedly higher when compared to control rats, which implies advanced heart edema. This effect was a result of increased production of ROS. 

However, we found no significant differences in the inflammatory parameters between the atria and ventricles, but differences were found in the oxidative parameters. The TBARS values differed in the LPS groups between the ventricles and atria, which indicates that the ventricles generated higher lipid peroxidation compared to the atria. 

In turn, with regards to H_2_O_2_ generation, it was higher in the atria than in the ventricles. 

Previous studies have reported that the concentration of proinflammatory mediators is higher in the left ventricle than in other parts of the rat heart. However, it is associated with chronic inflammation or prolonged exposure to oxidative stress, for example, in AF. In a failing heart, oxidative stress correlates with left ventricular dysfunction [44]. There is evidence that inflammation is involved in atrial remodeling in AF [45]. Our study presented a model of acute inflammation, which induces equally strong activation of proinflammatory factors in both the atria and ventricles. We suppose that differences would be more visible after longer exposure to oxidative stress.

The results of many experimental models correspond with the results we obtained in our study, in which we confirmed that ROS generated by oxidative stress increase the levels of oxidation markers. In one of these studies, the H_2_O_2_ level was increased in rat atria stimulated by a high pacing frequency [46]. These results suggest that a high concentration of H_2_O_2_ in the rat atria is associated with intracellular ROS formation in response to a high pacing frequency.

Proinflammatory factors adversely affect the cardiomyocyte structure, endothelium, and heart functions. Moreover, TNF-α activates the nuclear factor kappa B (NF-κB) of cardiomyocytes, which increases the expression of adhesion molecules and further promotes inflammatory injury to cardiomyocytes [47]. Chronic inflammation can lead to oxidative stress.

Other studies have indicated that TNF-α downregulates glutamate receptors and cardiomyocyte contractile proteins and causes cell death and apoptosis [48]. It can lead to the development of HF, which is associated with a loss of myocardial contractile forces and increased susceptibility to arrhythmias. 

Our data indicate that oxidative stress significantly inhibits SOD, which is possibly caused by a decreased concentration of this enzyme. This in turn is caused by enhanced lipid peroxidation under oxidative stress conditions. Similarly, other authors have indicated that elevated lipid peroxidation in oxidative stress may result from decreased activities of antioxidant enzymes [49].

In the present study, the decrease in the content of -SH groups after LPS administration also suggests an increased formation of ROS in the rat ventricles and atria. Similarly, another study revealed that reduced contents of the sulfhydryl group may be attributed to overproduction of ROS (superoxide) [50]. In another study, cadmium-induced oxidative damage increased levels of lipid peroxidation and structural alterations in the heart [51]. Furthermore, heart damage induced by cyclophosphamide decreased levels of glutathione, malondialdehyde (MDA), and calcium (Ca^+2^) in the heart [52]. In turn, the diminished level of GSH in cells increases oxidative stress in mitochondrial fractions and tissues. In our study, we observed decreased GSH levels and an increased GSSG concentration during oxidative stress (both in the ventricles and atria). Moreover, it was shown that administration of LPS decreased the intracellular ratio of GSH/GSSG, which indicates that LPS altered the antioxidant capacity and thiol redox state in the rat ventricles and atria.

It is well known that α-LA is a direct antioxidant produced from octanoic acid in mitochondria. Endogenous levels of plasma LA are reported to be 1–25 ng/mL in healthy humans, and so far, no clinical symptoms specific for lipoic acid deficiency in humans and animals are known [53]. α-LA is able to directly scavenge reactive oxygen species, and may provide antioxidant protection through transition metal chelation, and regeneration of reduced forms of some endogenous antioxidants (vitamin E and C and glutathione). The mechanism of α-LA’s action is related to modulation of the signaling transduction of several pathways, such as nuclear factor kappa-light-chain-enhancer of activated B cells (NF-kB) and mitogen-activated protein kinases (MAPKs) [3].

Reports from the literature show that α-LA can be used in various disorders related to oxidative stress [54,55,56].

In our experimental model, the antioxidant properties of α-LA were manifested by decreased inflammatory and oxidative parameters in the rat ventricles and atria. α-LA showed a higher effect on the ventricles in relation to the -SH and TBARS concentration. 

Furthermore, α-LA significantly restored the reduced GSH level and ameliorated the GSH/GSSG ratio, suggesting that the mechanism underlying α-LA’s action is a regeneration of GSSG to GSH. GSH plays an important role in the protection of vitamin E and C levels and is involved in an enzymatic detoxification reaction for ROS as a cofactor or coenzyme [57,58]. 

The results obtained in our study are in accordance with the works of other authors [59,60]. It was also shown that α-LA, via its anti-inflammatory properties, decreased TNF-α and IL-6 levels in many tissues, including cardiac tissue [61,62].

α-LA is considered to be a powerful antioxidant compared to other antioxidants, including vitamins A, E and C, and coenzyme Q10; flavonoids; and acetyl-L-carnitine. One study compared the effectiveness of α-LA with the effectiveness of vitamin C in NO-mediated vasodilation in diabetic patients. Parameters of lipid peroxidation were measured and correlated with endothelial function tests. α-LA was found to be a better antioxidant than vitamin C [63]. 

Some experimental studies have indicated a synergist effect of α-LA with other antioxidants. It has been shown that acetyl-L-carnitine and α-LA play an important role in normal mitochondrial function, and that reduced levels of these compounds are associated with increased mitochondrial oxidant production [64]. α-LA protected cardiac mitochondrial complexes from NO-induced damage. 

The authors of another study tested mitochondrial function in an acute model of endotoxemia and the effect of α-LA as a therapeutic strategy. It was shown that α-LA prevents oxidative stress and mitochondrial dysfunction [65].

**Figure 7:**
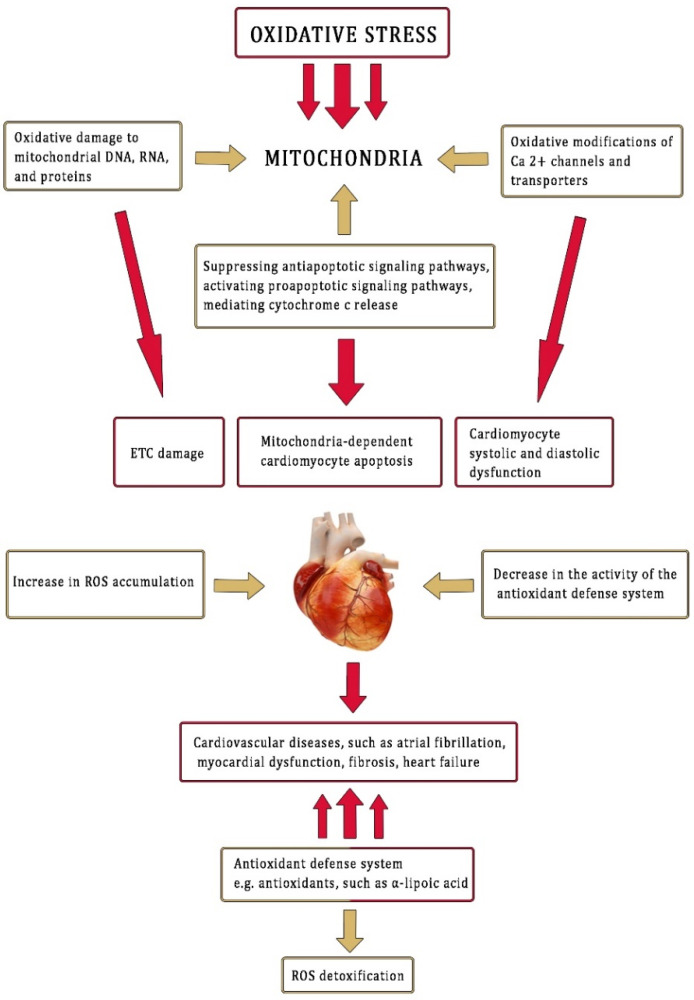
Effect of oxidative stress on cardiac damage. Adapted with permission from ref. [3]. Copyright 2015 Beata Skibska and Anna Goraca.

In the present work, the rat model of oxidative stress induced by LPS in the ventricles and atria was demonstrated (Figure 7). α-LA, as an antioxidant, was administered after the LPS challenge. α-LA reduced inflammatory and oxidative stress in the rat ventricles and atria with equal effectiveness. However, in this study, we did not assess histopathological changes in the heart nor proinflammatory and oxidant parameters in the blood, which represent limitations. Thus, further studies are necessary to compare other parameters related to this issue. 

## 5. Conclusions

Based on these studies, it can be concluded that α-LA is an ideal antioxidant in the prevention of oxidative stress development in the ventricles and atria because it alleviates lipid peroxidation, scavenges free radicals, and enhances the synthesis of antioxidants containing -SH groups and GSH. 

However, further research is needed to explain the mechanism of action of α-LA in oxidation-stressed ventricular and atrial tissues.

## Figures and Tables

**Table 1:** Changes in the HW (heart weight)/BW (body weight) ratio in the control, LA, LPS, and LPS + LA groups.

Group	HW/BW (mg/g)
Control	2.63 ± 0.17
LA	2.81 ± 0.20
LPS	3.68 ± 0.32 *
LPS + LA	2.97 ± 0.29 #

* *p* < 0.01 vs. control, LA; # *p* < 0.001 vs. LPS. Values are expressed as mean ± S.E.M., *n* = 8 in each group.

**Table 2:** Ventricles status of glutathione metabolism in the control, LA, LPS, and LPS + LA groups.

Parameters	Control	LA	LPS	LPS + LA
Total GSH (µM)	89.6 ± 5.03	82.7 ± 16.9	14.82± 2.2 ***,##	129.63 ± 16.4 *
GSH (µM)	72.8 ± 6.9	59.2 ± 5.8	5.96 ± 2.4 ***	101.52 ± 5.9 **
GSSG (µM)	22.5 ± 4.2	6.76 ± 0.7	20.4 ± 2.3 &&	20.83 ± 2.1
GSH/GSSG (redox status)	3.7 ± 1.2	7.38 ± 0.6	0.31 ± 0.12 #,&	4.87 ± 0.35 **

* *p* < 0.01 vs. LPS; ** *p* < 0.001 vs. LPS; *** *p* < 0.01 vs. control; # *p* < 0.05 vs. control; ## *p* < 0.02 vs. LA; & *p* < 0.001 vs. LA; && *p* < 0.01 vs. LA. Values are expressed as mean ± S.E.M., *n* = 8 in each group.

**Table 3:** Atria status of glutathione metabolism in the control, LA, LPS, and LPS + LA groups.

Parameters	Control	LA	LPS	LPS + LA
Total GSH (µM)	85.9 ± 4.923	79.8 ± 17.1	13.9 ± 2 ***,##	119.92 ± 15.9 *
GSH (µM)	71.2 ± 6.5	58.3 ± 5.2	5.4 ± 2.1 ***	99.82 ± 5.7 **
GSSG (µM)	20.9 ± 3.9	6.02 ± 0.6	19.9 ± 2.3 &&	20.23 ± 2
GSH/GSSG (redox status)	3.5 ± 1.1	6.96 ± 0.5	0.29 ± 0.11#,&	4.06 ± 0.34 **

* *p* < 0.01 vs. LPS; ** *p* < 0.001 vs. LPS; *** *p* < 0.01 vs. control; # *p* < 0.05 vs. control; ## *p* < 0.02 vs. LA; & *p* < 0.001 vs. LA; && *p* < 0.01 vs. LA. Values are expressed as mean ± S.E.M., *n* = 8 in each group.

## Data Availability

Data is contained within the article.
